# A Multidimensional Approach to Assessing Factors Impacting Health-Related Quality of Life after Pediatric Traumatic Brain Injury

**DOI:** 10.3390/jcm12123895

**Published:** 2023-06-07

**Authors:** Nicole von Steinbuechel, Ugne Krenz, Fabian Bockhop, Inga K. Koerte, Dagmar Timmermann, Katrin Cunitz, Marina Zeldovich, Nada Andelic, Philine Rojczyk, Michaela Veronika Bonfert, Steffen Berweck, Matthias Kieslich, Knut Brockmann, Maike Roediger, Michael Lendt, Anna Buchheim, Holger Muehlan, Ivana Holloway, Laiene Olabarrieta-Landa

**Affiliations:** 1 Institute of Medical Psychology and Medical Sociology, University Medical Center Göttingen, Waldweg 37A, 37073 Göttingen, Germany; 2 cBRAIN/Department of Child and Adolescent Psychiatry, Psychosomatics and Psychotherapy, LMU University Hospital, Ludwig-Maximilian University, Nussbaumstrasse 5, 80336 Munich, Germany; 3 Research Centre for Habilitation and Rehabilitation Models and Services (CHARM), Department of Health and Society, University of Oslo, 0316 Oslo, Norway; 4 Department of Physical Medicine and Rehabilitation, Oslo University Hospital, 0424 Oslo, Norway; 5 Department of Pediatric Neurology and Developmental Medicine, LMU Center for Development and Children with Medical Complexity, Dr. von Hauner Children’s Hospital, LMU University Hospital, Haydnstr. 5, 80336 Munich, Germany; 6 Specialist Center for Paediatric Neurology, Neurorehabilitation and Epileptology, Schoen Klinik, Krankenhausstraße 20, 83569 Vogtareuth, Germany; 7 Department of Paediatric Neurology, Hospital of Goethe University, Theodor-Stern-Kai 7, 60590 Frankfurt am Main, Germany; 8 Interdisciplinary Pediatric Center for Children with Developmental Disabilities and Severe Chronic Disorders, Department of Pediatrics and Adolescent Medicine, University Medical Center, Robert-Koch-Str. 40, 37075 Göttingen, Germany; 9 Department of Pediatric Intensive Care Medicine and Neonatology, University Hospital Muenster, Albert-Schweitzer-Campus 1, 48149 Muenster, Germany; 10 Neuropediatrics, St. Mauritius Therapeutic Clinic, Strümper Straße 111, 40670 Meerbusch, Germany; 11 Institut für Psychologie, Universität Innsbruck, Innrain 52 f, 6020 Innsbruck, Austria; 12 Department of Health and Prevention, University of Greifswald, Robert-Blum-Str. 13, 17487 Greifswald, Germany; 13 Departamento de Ciencias de la Salud, Universidad Pública de Navarra, Campus de Arrosadía, 31006 Pamplona, Spain; 14 Instituto de Investigación Sanitaria de Navarra (IdiSNA), 31008 Pamplona, Spain

**Keywords:** child, adolescent, traumatic brain injury, post-concussion symptoms, depression, anxiety, health-related quality of life

## Abstract

In the field of pediatric traumatic brain injury (TBI), relationships between pre-injury and injury-related characteristics and post-TBI outcomes (functional recovery, post-concussion depression, anxiety) and their impact on disease-specific health-related quality of life (HRQoL) are under-investigated. Here, a multidimensional conceptual model was tested using a structural equation model (SEM). The final SEM evaluates the associations between these four latent variables. We retrospectively investigated 152 children (8–12 years) and 148 adolescents (13–17 years) after TBI at the recruiting clinics or online. The final SEM displayed a fair goodness-of-fit (SRMR = 0.09, RMSEA = 0.08 with 90% CI [0.068, 0.085], GFI = 0.87, CFI = 0.83), explaining 39% of the variance across the four latent variables and 45% of the variance in HRQoL in particular. The relationships between pre-injury and post-injury outcomes and between post-injury outcomes and TBI-specific HRQoL were moderately strong. Especially, pre-injury characteristics (children’s age, sensory, cognitive, or physical impairments, neurological and chronic diseases, and parental education) may aggravate post-injury outcomes, which in turn may influence TBI-specific HRQoL negatively. Thus, the SEM comprises potential risk factors for developing negative post-injury outcomes, impacting TBI-specific HRQoL. Our findings may assist healthcare providers and parents in the management, therapy, rehabilitation, and care of pediatric individuals after TBI.

## 1. Introduction

The estimated incidence of traumatic brain injuries (TBI) is more than 50 million individuals worldwide annually and around 262 per 100,000 inhabitants in Europe [1]. Independently of TBI severity, children and adolescents may experience a variety of impairments following TBI that negatively affect their functioning, participation, and quality of life. Post-TBI outcomes are also frequently characterized by emotional disorders [2,3], post-concussion symptoms (PCS) [4], and prolonged functional recovery. More specifically, increased levels of anxiety and depression after TBI have been reported in children with a history of TBI [5]. Pre-morbid psychiatric impairment has also been associated with more severe depressive symptomatology [6] and a higher number of PCS [7] after TBI. Depression [2] and PCS [4] have also been linked to current poor mental health in family members.

Regarding socio-demographics, a lower socioeconomic status (SES) is related to a higher frequency of PCS [8] and an increased risk of developing depression after TBI [6,9]. However, findings concerning the impact of age on PCS and anxiety are inconclusive. Some studies have noted more severe anxiety [10] and PCS [11,12] in older children, while other studies show the opposite [3,13,14]. Regarding sex, girls report more PCS compared with boys after TBI [13,15], but no relevant differences are reported concerning mental health [16].

Concerning injury characteristics, higher TBI severity has a negative impact on various outcomes after pediatric TBI, including functional recovery [17], cognitive performance [18], social functioning [19], and HRQoL [17]. It is also correlated with an increased prevalence of anxiety disorders [20], with a less robust association with depression [21]. Frontal lobe lesions have been linked to depression [2] and anxiety [3,16,22], as has the presence of intracranial abnormalities and more severe PCS in the acute phase of pediatric TBI [8]. Cognitive impairments after TBI were linked to the depth [23] as well as the number and volume of brain lesions [24].

The prevalence of post-injury outcomes, e.g., pediatric PCS, ranges from 29.3% to 52% depending on the diagnostic criteria, assessment tools, and patient populations [7]. They may persist for weeks or become chronic [25], interfering with normal school functioning [26]. At the subclinical and clinical levels, depression and anxiety range from 1.6% to 60% [3,27]. They are often highly comorbid [23], persisting over many years beyond childhood [28]. In addition, behavioral difficulties can persist five years after TBI [29].

Previous research has mostly assessed outcomes after pediatric TBI unidimensionally and relied on parental reporting, which may lead to rater bias [30], misdiagnoses, or a failure to detect individuals at risk of developing emotional [31] and psychosocial problems [32] and impaired functional recovery [33].

The multidimensional assessment of HRQoL represents a step towards capturing a more comprehensive patient-centered picture of the consequences of TBI in several domains of life. It encourages collecting children’s and adolescents’ self-reports after TBI and is well suited to capture the complex nature of TBI from a subjective perspective. This multidimensional construct covers individuals’ well-being in the domains of emotional, cognitive, physical, social, and daily life functioning and can be measured using generic or disease-specific instruments [34]. Generic tools may not be particularly sensitive to specific aspects and sequelae of a defined disease or health condition. Also, in the field of TBI, the disease-specific HRQoL measurement can be more sensitive to the impact of TBI in the respective life domains [35]. Reduced HRQoL has been consistently reported in children after TBI [36] until young adulthood [37]. Moreover, poorer HRQoL has been linked to depression [6,21] and PCS [38], persisting up to 12 months after TBI [39]. Lower physical and cognitive HRQoL has also been associated with the length of functional recovery and the initial severity of TBI symptoms in young athletes [40].

The multitude of impaired outcomes after TBI underlines the need for a comprehensive multidimensional outcome assessment [34,41] to broadly capture the effect of pediatric TBI. The resulting conclusions could support the development of early personalized clinical interventions and care to prevent, manage, and ameliorate adverse outcomes in children and adolescents after TBI and provide important information for the affected families. However, an empirical operationalization of a comprehensive multidimensional biopsychological model [41,42] of factors influencing post-TBI outcome and their effects on HRQoL is missing so far in the field of pediatric TBI.

The aim of this pediatric TBI study is to test a multidimensional conceptual model investigating the relationship between pre-injury and injury-related characteristics and post-injury outcomes (depression, anxiety, PCS, functional recovery), as well as their impact on TBI-specific HRQoL. In the clinical context, a multidimensional model offers the possibility of a truly comprehensive outcome evaluation. Based on this, guidance is provided on how to treat a pediatric individual after TBI with specific pre-injury and injury-related characteristics, e.g., identifying those that should receive priority treatment compared to low-risk patients.

## 2. Materials and Methods

### 2.1. Study and Study Participants

For this retrospective multicenter study, 300 participants with a history of TBI from the German-language context were included, meeting the following criteria: (a) 8–17 years of age; (b) a diagnosis of TBI at least 3 months but no more than 10 years before study participation; (c) a formal Glasgow Coma Scale (GCS) score or TBI severity recorded; (d) outpatients (or about to resume inpatient treatment); and (e) able to understand and answer the questions. Participants were excluded if they: (a) were currently in a vegetative state (i.e., minimally conscious state according to the Coma Recovery Scale-Revised); (b) had spinal cord damage; (c) had severe mental illness before TBI (e.g., psychosis, autism, etc.); (d) had epilepsy before TBI; (e) had a disease leading to death; or (f) had very severe poly-trauma.

Further clinical details, such as loss of consciousness, amnesia, requirement for ventilation, neurosurgical intervention, presence of a lesion on imaging, resuscitation, nausea/vomiting, and post-traumatic epilepsy, were also collected. This information was used to describe the TBI in the absence of a GCS score. An open-ended text field completed for other post-TBI symptoms contained post-TBI agitation and/or delirium, reported in eight children (2.7%).

Participants who met the inclusion criteria were contacted by postal mail and invited to participate in the study. Participants and their families were informed about the research aim and procedure before giving their written consent. All participants and their parents signed the informed consent and medical records release form. Participants were recruited from January 2019 until January 2022 from hospital registries in Germany.

A sample size estimation for the project was performed beforehand for all planned analyses assuming equal numbers of factors in all age groups indicating the need for at least 140 subjects per age group [43].

### 2.2. Ethical Approval

The study was conducted in accordance with all relevant laws of Germany including but not limited to the ICH Harmonized Tripartite Guideline for Good Clinical Practice (“ICH GCP”) and the World Medical Association Declaration of Helsinki (“Ethical Principles for Medical Research Involving Human Subjects”). The study (application number 19/4/18) was approved by the Ethics Committee of the University Medical Center in Göttingen.

### 2.3. Procedure

The examinations were administered face-to-face at the recruiting clinics (74.7%) or online (25.3%). Eligible and consenting participants, their parents, and investigators provided the data. Clinical data were retrieved from medical records by clinicians and psychologists and transcribed into the patient case report forms (CRFs). Parents filled out paper and pencil CRFs at the recruiting centers or sent these back via postal mail. Age-appropriate CRF booklets were administered to two different age groups (8–12 and 13–17 years of age). The age split was based on the biopsychosocial developmental model of Havighurst [44] and recent pediatric HRQoL research studies, which describe, for example, the development of the Pediatric Quality of Life Inventory [45].

### 2.4. Methods

**Figure 1:**
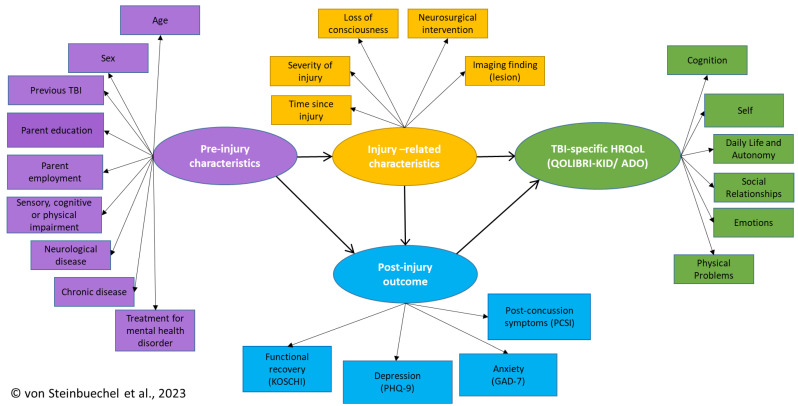
Multidimensional conceptual model for testing factors impacting TBI-specific HRQoL.

A comprehensive multidimensional conceptual model (Figure 1) inspired by the depiction of a trajectory analysis of PCS [41] was tested empirically in this study using a structural equation measurement modeling (SEM) approach. Four groups of factors were used to construct the final empirical model: pre-injury and injury-related characteristics, post-injury outcomes, and disease-specific HRQoL.

#### 2.4.1. Pre-Injury Characteristics

The conceptual recursive model contained the following pre-injury data: age (from medical records) and sex, parental education, employment status, previous TBIs, presence of neurological disease, presence of chronic disease, treatment for mental health disorder prior to the injury, any sensory, cognitive, or physical impairment (reported by parents). Pre-injury characteristics form a latent variable that takes into account the effect of chronic diseases, as well as age, sex, etc. This allows the influence of, for example, chronic diseases to be controlled for, since pre-injury characteristics are related to the rest of the latent variables in the model, including the post-injury outcome.

#### 2.4.2. Injury-Related Characteristics

Clinical data retrieved from the medical notes included the severity of the injury, presence of any lesion detected by CT or MRI, presence of retrograde amnesia, LOC, and neurosurgical intervention following injury, and time since injury.

#### 2.4.3. Post-Injury Outcomes

Functional recovery was determined using clinical ratings using the Kings Outcome Scale for Childhood Head Injury (KOSCHI, assessed by investigators during the interview) [46]; PCS was measured by administering the Post-Concussion Symptom Inventory (PCSI, self-completed by patients) [47]. Major depression disorder was assessed using the Patient Health Questionnaire 9 (PHQ-9) [48] completed by parents, as was the Generalized Anxiety Disorder-7 (GAD-7) [49]. Self-reported TBI-specific HRQoL was captured using the Quality of Life after Traumatic Brain Injury in Kids/Adolescents (QOLIBRI-KID/ADO) questionnaire [50]. Details of the instruments can be found in Appendix A—Instruments and measures.

### 2.5. Statistical Considerations

During the process of the model building based on the multidimensional conceptual model (Figure 1), the following continuous variables were used: age, time since injury, PCSI, PHQ-9, GAD-7, and QOLIBRI-KID/ADO. In addition, the following dichotomized variables were used: sex (0—Female, 1—Male), parental education (0—University, 1—Other than university), parental employment (0—Employed >35 h per week, 1—Other than >35 h per week), previous TBI (0—None, 1—One or more), TBI severity (0—Mild, 1—Moderate or severe), and KOSCHI (0–3a, 3b, 4a, 4b; 1–5a, 5b). The remaining variables (i.e., prior cognitive, sensory, or physical impairment; the presence of neurological disease or chronic disease; treatment for mental health disorder prior to injury; the presence of any lesion detected by CT or MRI; the presence of retrograde amnesia; LOC; neurosurgical intervention following injury) were dichotomized to either 0 (No) or 1 (Yes).

Descriptive statistics were calculated to summarize all variables, subscales, and total scores of the measures by age group: KIDs (8–12 years), ADOs (13–17 years), and overall. Means, standard deviations, medians, and ranges were used for continuous variables, and counts and percentages for categorical variables. Continuous data were assessed for normality. In the absence of normality, data were transformed using the appropriate transformation (e.g., square root transformation for zero-inflated data or small values, or log transformation for positively skewed data).

Differences between the two age groups were investigated using parametric and non-parametric tests, depending on the data distribution. Differences between SES, measured in terms of parental education, and HRQoL, PCS, anxiety, and depression symptoms (assessed with the QOLIBRI-KID/ADO, PCSI, GAD-7, and PHQ-9) were also examined using non-parametric Kruskal–Wallis tests.

### 2.6. Missing Data

Missing data for self-reported measures were expected at the item or scale level. For missing data at the item level, prorating was performed for all self-reported measures if a minimum number of items had been completed. For PCSI, if the number of missing items was one or two, prorating was used to calculate a score [51]. If up to one-third of the values of the PHQ-9 and the GAD-7 were missing, they were substituted with the mean score of the non-missing items when calculating a total score. For the QOLIBRI-KID/ADO, prorating was applied if up to one-third of subscale values were missing.

To fully utilize all the available information from the study data set in the presence of missing values, the full information maximum likelihood (FIML) method was used to handle missing data on demographics and clinical characteristics and in cases where the entire questionnaires were missing. All analyses were conducted using SAS software, version 9.4, using the PROC CALIS procedure.

### 2.7. Path Analysis

The following latent factors were integrated into the path analysis: pre-injury and injury-related characteristics, post-injury outcome, and TBI-specific HRQoL. The indicator variables defining each latent variable are listed in the multidimensional conceptual model section (see Figure 1).

A two-step procedure was performed [52] in building the final empirical SEM, starting with the initial measurement model, its validation, and revision as necessary, followed by modification of the measurement model to develop the empirical model (including revision if needed). The use of SEM was confirmatory. The data were not nested, and all observations were independent.

First, the measurement model for testing the strength of the relationship between the observed variables and the factors was created using a confirmatory analysis. Path analysis determined whether the model accounted for the relationships observed in the sample. It was also checked whether the direction of the relationships between the observed variables in the conceptual model was valid for the sample. The covariance was estimated to relate each latent variable to each other. Every latent variable contained at least four manifest variables. Furthermore, a composite reliability index and variance extracted estimates were calculated for each latent factor. A composite reliability index assesses the degree of reliability with which the latent factor explains the variance in indicator variables. This is an indicator of the internal consistency of indicators within a given factor [52]. A reliability index of 0.70 is considered to be acceptable [53]. Variance extracted estimates, which should be above 0.49 [54], assess the amount of variance captured by latent factors in relation to the variance attributable to measurement error.

In the second step, the measurement model was modified to create an empirical model based on the original conceptual model. The model was tested and revised until a theoretically and statistically acceptable model was obtained.

Recommendations for reporting the results of data analyzed using structural equation modeling [55] were followed, as were those for the range of fit statistics considered for the model fit. As reliance on a single fit criterion is not recommended [56], a range of global fit statistics was used (desirable values in parentheses) as follows:

- The ratio of chi-square statistics/degrees of freedom (*χ*^2^/df < 3) [57];
- The root mean square error of approximation (RMSEA; mediocre within 0.08–0.10, fair within 0.05–0.08, ideal fit < 0.05), as well as 90% confidence limits (adequate within 0.0001–0.090, ideal within 0.0001–0.054) [58];
- Standardized root mean square residual (SRMR; ideal < 0.055) [58];
- Goodness-of-fit index (GFI) and comparative fit index (CFI) (> 0.90 acceptable, > 0.94 very good fit) [58].

All indices were checked and the model was modified until it was acceptable, until the factors made theoretical or clinical sense and further modifications were not appropriate. All changes from the original conceptual model were documented. Standardized graphical model solutions were presented.

## 3. Results

### 3.1. Participants

**Figure 2:**
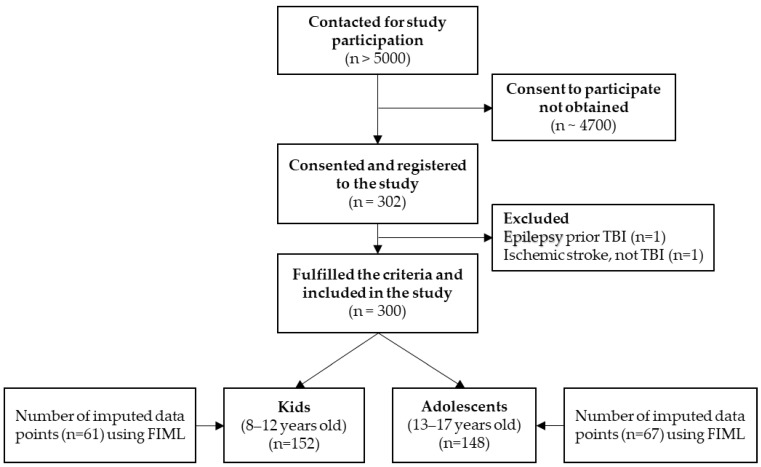
Flow chart of study participants and number of imputed items in the SEM building. Note. FIML = Full Information Maximum Likelihood; Sociodemographic and injury-related characteristics of the two pediatric TBI age groups are listed separately and overall in Table 1 as well as significant group differences. Missing data points can be found in Table 1, Table 2 and Table 3.

A total of 300 children and their parents participated in the study, with 152 in the KID group and 148 in the ADO group; see the flow chart in Figure 2. The participants’ pre-injury characteristics are summarized in Table 1. There were more males (59.3%), a high proportion of participants lived in a family where at least one parent worked more than 35 h per week (87.3%), and in 63.0% of the families, at least one parent was educated to university level. The majority of participants reported no previous TBI (81.7%), had no previous cognitive, sensory, or physical impairment (71.3%), prior neurological disease (91.3%), chronic disease (73.0%), or treatment for mental health disorders (89.0%). There was no evidence of statistically significant differences in pre-injury characteristics between the KID and ADO groups.

In terms of injury characteristics, a large proportion of participants had experienced a mild TBI (71.7%). Lesions in the CT or MRI were reported for 28.7% of participants, 25.0% of individuals reported retrograde amnesia and 31.7% experienced LOC. Neurosurgical interventions were necessary for 17.0% of the patients. The time since injury ranged from 0 to 10 years, as per eligibility criteria, with a median of 3.7 years in the KID group and 4.5 years in the ADO group. Statistically significant differences were determined between age categories concerning retrograde amnesia and LOC following injury; a higher proportion of participants in the ADO group reported amnesia and LOC compared with those in the KID group.

Summaries of the post-injury outcome measures are presented in Table 2. PCSI scores were log-transformed due to severe skewness and then standardized for building the model because the scales for the KID and ADO groups were different. The square root transformation was used for the PHQ-9 and GAD-7 scores due to the data being positively skewed. The majority of participants (89.7%) reported good or complete recovery (categories 5a or 5b, respectively) as measured by the KOSCHI. The mean post-injury PCSI score for ADOs was 19.8 points (of a maximum of 75 points) and 5 points for KIDs (out of 27 points). Overall, 69.3% of the parents reported no or minimal anxiety, and 62.3% of the parents reported no or minimal depression in their children.

QOLIBRI-KID/ADO self-reported scores by subscale and overall are summarized in Table 3. There were statistically significant subscale differences between the KID and ADO groups in terms of the total score, cognition, self, daily life and autonomy, and social subscales. The ADO group reported lower HRQoL in all these subscales compared to the KID group.

Scores for depression, anxiety, post-concussion symptoms, and HRQOL differentiated by parental education are listed in Table 4. There was no evidence of statistically significant differences between QOLIBRI-KID/ADO Total and PCSI self-reported scores concerning parental education. However, differences between parent-reported GAD-7 and PHQ-9 scores and parental education were statistically significant. Children’s GAD-7 and PHQ-9 scores were lower in households with at least one parent with a university education compared with households with parents with other than a university education.

### 3.2. Missing Data

No systematic pattern was observed in the structure of the missing data. In all but the PCSI variable, there were 5% or fewer values missing in the indicator variables. We considered data to be missing completely at random. A total of 13.7% of overall PCSI scores were missing for the participants recruited at the beginning of the study due to the PCSI questionnaire data not being collected at that time.

### 3.3. Overview of the Analysis

#### 3.3.1. The Initial and the Revised Measurement Model

The initial measurement model comprised all variables and latent factors as stated in the multidimensional conceptual model (see Figure 1).

The goodness-of-fit statistics for the initial measurement model are presented in Table 5. The measurement model was re-specified to contain only indicator variables with significant factor loadings (*p*-value < 0.01); those whose factor loadings were not significant were excluded: sex, employment, previous TBI, and time since injury.

#### 3.3.2. The First and Revised Empirical SEM

With a sample of 300 participants and 166 degrees of freedom (df), the statistical power of the model was approximately 0.99. The global fit statistics and model are shown in Table 5. The goodness-of-fit values in the first empirical SEM were similar to those in the revised measurement model for the CFI with a value of 0.83, the GFI 0.87, the SRMR 0.09, and the RMSEA 0.08. The 90% RMSEA confidence limits were acceptable, with a 90% CI [0.068, 0.085]. All the goodness-of-fit values suggest a fair model fit.

The absolute value of the t-statistics for each factor loading and path coefficient between pre-injury characteristics and post-injury outcome, and post-injury outcome and TBI-specific HRQoL, exceeded 1.96. The path coefficient linking the latent constructs of pre-injury characteristics and injury-related characteristics and the path coefficients from injury-related characteristics to the post-injury outcome and TBI-specific HRQoL displayed a t-value smaller than 1.96, suggesting there is evidence that these paths are not statistically significant. The standardized path coefficient for the path from pre-injury to injury-related characteristics was small, at 0.14. The standardized path coefficients from injury-related characteristics to post-injury outcome (0.09) and TBI-specific HRQoL (0.02) were also small. This indicates that the model does not account for the relationship between these latent factors. Therefore, in the revised empirical SEM, the weakest path between injury-related characteristics and TBI-specific HRQoL was removed with no change to the revised measurement model (Table 5).

The chi-square difference value comparing the first empirical model with the revised measurement model was 3.8 (df = 1, *p* = 0.051), suggesting that the empirical model provides an adequate fit with the data and is not significantly worse than the fit of the revised model (Table 5).

Reliability estimates for the variables in the final SEM and the variance extracted estimates are listed in Table 6. The values of the composite reliability for pre-injury characteristics (reliability r = 0.49) and post-injury outcomes (r = 0.56) are below 0.70. Injury-related characteristics and TBI-specific HRQoL fulfill this requirement. The variance extracted for the injury-related characteristics was the largest, at 57%. Overall, the average variance estimate was 0.39 across the four factors. The standardized factor loadings for the indicators measuring the relevant latent factors were statistically significant, suggesting the convergent validity of the indicators; however, the magnitude of some factor loadings was small (below 0.4); the standardized absolute values of factor loadings ranged from 0.22 to 0.92; none of these were negligible (Table 6).

The findings suggest that the amount of variance captured by the factors of pre-injury characteristics and post-injury outcome is smaller compared with the variance due to random measurement error.

**Figure 3:**
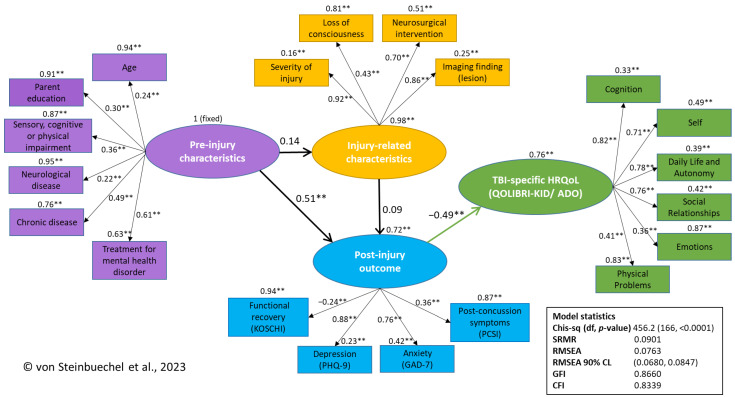
Final empirical SEM path diagram showing the final parameter estimates and their statistical significance. Note. Significant estimates are flagged with an asterisk (** *p* < 0.01). The pathway from Post-injury outcome to TBI-specific HRQoL in green: higher scores in GAD-7, PHQ-9, and PCSI are negatively associated with TBI-specific HRQoL (lower QOLIBRI-KID/ADO scores).

The final revised SEM contains the following latent factors and indicator variables (in brackets): pre-injury characteristics (age, education, composite of sensory, cognitive, or physical impairment, neurological disease, chronic disease, and treatment for mental health disorder), post-injury characteristics (severity of injury, LOC, neurosurgical intervention, and imaging finding), post-injury outcome (KOSCHI, PHQ-9, GAD-7, and PCSI), and TBI-specific HRQoL (QOLIBRI-KID/ADO) (Figure 3). The model fit indices suggest a fair model fit with values of SRMR (0.09), RMSEA (0.08), 90% CI [0.068, 0.085], GFI (0.87), and CFI (0.83) (Table 5).

## 4. Discussion

The present study aimed to assess a multidimensional conceptual framework of factors impacting outcomes after TBI, inspired by an illustration of the development of PCS [41]. From this framework model, we developed an SEM investigating pre-injury, injury-related characteristics, and post-injury outcomes impacting HRQoL after pediatric TBI.

Before examining the final model, differences in pre-injury and injury-related characteristics between the KID and the ADO groups were explored. The groups only differed concerning retrograde amnesia, where a higher proportion of ADOs reported retrograde amnesia compared to the KIDs. This may be because more adolescents have suffered LOC. Regarding recovery status, good or complete recovery was reported more frequently than moderate or severe disability. This pattern of predominantly good recovery after pediatric TBI is consistent with previous findings [59] and maybe mainly explained by the composition of the sample, with over 71% of participants having experienced a mild TBI.

Parental education and lower SES have been linked to emotional and behavioral outcomes, including anxious/depressive symptomatology [6,60], and higher PCS [8]. In our sample, there was evidence of statistically significant differences concerning children’s anxiety and depressive symptomatology in association with parental education. Parents with a university education reported lower anxiety and depression scores for their children compared with those with no university education. A lower level of parental education is often associated with a lower SES, representing a risk factor for mental health problems [61]. Therefore, our results are in line with previous studies.

Significant differences between both age groups were also found regarding disease-specific HRQoL after TBI, with adolescents reporting lower scores for the subscales Cognition, Self, Daily life and autonomy, and Social relationships compared with the younger group. The literature shows that age at injury is strongly associated with HRQoL after pediatric TBI; children who are younger at the time of injury have better HRQoL than older ones [21]. As previously suggested [21], early TBI may cause a less dramatic life change for children, as some of them may not even remember life before the TBI, unlike adolescents.

Several steps had to be performed to reach the final SEM (Figure 3) based on the conceptual multidimensional assessment model (Figure 1) predicting the relationships between the four latent constructs (pre-injury, injury-related characteristics, post-injury outcomes, and TBI-specific HRQoL). In the following paragraphs, our hypotheses concerning the associations will be discussed in detail.

Variables without statistically significant loadings (parental employment, sex, previous TBI, and time since injury) with respect to the assumed latent constructs were excluded from the final SEM.

The need to exclude the parental employment variable contrasts with findings in the literature indicating that SES has an impact on a child’s ability to recover from injury and post-injury therapy [62]. Lower family income has been associated with a higher percentage of pediatric TBI mortality [63] and higher levels of emotional difficulties and conduct problems at 12 months post-injury [64]. The lack of employment variability may explain why this variable was not included in the SEM model; the majority of households had at least one parent employed for more than 35 h a week (87.3% of households in our sample), compared to households where parents worked less (10.3%).

Sex is also a controversial variable regarding outcomes after TBI. In our study, it did not display a significant factor loading on injury-related characteristics when inspecting the initial SEM. There are several preclinical studies supporting the importance of sex differences in response to pediatric TBI. However, TBI outcomes are also likely to depend on many other factors, such as age at injury, mechanisms of injury, and time post-injury [65].

Previous TBI and time since injury were also excluded from the final SEM as they were not significant. Only a small proportion of children had reported prior TBIs.

The relationship between injury-related characteristics and pediatric post-TBI outcomes remains relatively unclear [59]. In particular, it is debatable whether depression and anxiety are primary or secondary outcomes following a pediatric TBI. Unfortunately, our model cannot disentangle this relationship. However, the impact of pre-injury characteristics is strong. Thus, some of the post-injury outcomes could be interpreted as consequences of significant lifestyle changes after TBI. Brain lesions (mostly in the frontal lobe) have been related to depression and anxiety symptomatology after pediatric TBI [3,16]. However, this was not observed in our study, which could be due to the lack of lesion findings in 68% of the participants and no neurosurgical intervention after TBI in 82.3%. Previous studies have reported injury severity to be associated with personality changes or anxiety disorders [20] and functional outcome [17]; however, in our study, the majority of children recovered well (89.7%), and no or minimal anxiety (62.3%) or depression (69.3%) was reported.

Nevertheless, the final SEM suggests the importance of post-injury outcomes as part of the pathway between pre-injury characteristics and disease-specific HRQoL. Several pre-injury characteristics (children’s age, sensory, cognitive, or physical impairments, neurological and chronic diseases, and parental education) may aggravate post-injury outcomes (including anxiety and depression symptomatology, PCS, and recovery status in our model), which in turn may influence TBI-specific HRQoL negatively. Previous studies have consistently described significant relationships between children’s pre-injury psychiatric disorders and depressive post-injury symptomatology [6,42,66] and post-injury PCS [7,8]. Based on the factor loadings in our model, we recommend considering the presence of neurological and chronic disease, as well as pre-injury mental health issues, as part of the pre-injury characteristics of children in understanding the factors affecting post-TBI outcomes and TBI-specific HRQoL.

The literature in the field of pediatric TBI is not clear concerning the relationship between injury-related characteristics and HRQoL, e.g., TBI severity has been inconsistently associated with generic HRQoL [21]. In our first SEM, we observed only a weak relationship between injury-related characteristics and disease-specific HRQoL, which was not statistically significant. Therefore, this pathway was excluded from the final SEM, possibly because our sample consisted mainly of individuals after mild TBI, where most injury-related factors probably had minimal if any, impact on the outcome.

However, we observed a moderately strong negative association between post-injury characteristics and TBI-specific HRQoL. This finding confirms observations from previous studies that have systematically reported a link between depression [6], anxiety [26], PCS [39], and functional recovery [67] with HRQoL in pediatric TBI.

In summary, according to our final SEM (Figure 3), the relationship between pre-injury and injury-related characteristics is weak. Post-injury outcomes are moderately impacted by pre-injury characteristics and weakly by injury-related characteristics. Finally, TBI-specific HRQoL is expected to be directly affected by post-injury outcomes. The average amount of variance explained (39%) is reasonable, suggesting that the factors that are almost always available in medical records or can be obtained through additional screening can be considered when assessing individual HRQoL after pediatric TBI.

### 4.1. Limitations

This study has several limitations. Firstly, pre-injury and post-injury latent variables presented relatively low composite reliability (r = 0.49 and r = 0.56, respectively). The weak reliability of the pre-injury characteristics (Table 6) may be because our sample was biased by self-selection. The majority of children after TBI had no previous TBI, cognitive, sensory, or physical impairments, neurological diseases, or chronic diseases. Most parents achieved a tertiary level of education and full-time employment. Therefore, not much variability was present in the data. Regarding the post-injury outcome latent variable, the low composite reliability may also be linked to the different procedures used in data collection, as the data sources were investigators (KOSCHI score), parents (PHQ-9 and GAD-7), and KIDs and ADOs self-reports (PCSI).

Secondly, prior literature has described other variables that may be involved in post-TBI outcomes, such as a family history of depression/anxiety [2] and biomarkers [68], which were not included in this study. Moreover, in a recent study, the presence of agitation and delirium in the post-TBI period was linked to a higher risk of post-traumatic cognitive impairment associated with increased disability and long-term cognitive impairment in pediatric populations [69]. We recommend that future studies look at disability after pediatric TBI and consider including these variables, also from a long-term perspective.

The integration of detailed cognitive outcomes in the current SEM would have gone beyond the scope of this study. However, these are being implemented in our ongoing re-validation of our model in a different pediatric sample after TBI. Although we tried hard to include all severity levels, most of the patients had experienced a mild TBI. Thus, our results could differ for those who faced more severe TBI.

Also, the fact that the range of time since injury was broad (0–10 years) may imply that the recovery of children might already have been completed several years after the TBI. Further studies are needed, including a shorter post-TBI time period, to better determine the impact of injury-related characteristics on outcomes and investigate their longitudinal development and trajectories of multidimensional recovery.

### 4.2. Implications

The identification of influential factors for recovery, amelioration of TBI-specific HRQoL after pediatric TBI, and their interrelationships impacting the development of evidence-based interventions is still limited. In our study, pre-injury characteristics appear to play a more important role in predicting post-injury outcomes than injury-related ones; they may influence TBI-specific HRQoL through their impact on post-injury anxiety, depression, PCS, and recovery status. The results of our final SEM offer support to clinicians and researchers by complementing their evaluation of patients and ameliorating clinical management, care, and rehabilitation after pediatric TBI. Early psychological therapy and, where indicated, medical treatment of depression, anxiety, and PCS arise as potential targets for improving HRQoL following pediatric TBI [70].

## 5. Conclusions

This study investigated the impact of pre-injury and injury-related characteristics, as well as post-TBI outcomes (anxiety, depression, PCS, and functional recovery), on the disease-specific HRQoL of individuals after pediatric TBI. To our knowledge, the current study is the first to integrate a variety of manifest and latent variables concerning TBI in a single multidimensional SEM. Injury-related characteristics were only weakly associated with post-injury outcomes and not directly with TBI-specific HRQoL. Contrary to our hypothesis, pre-injury characteristics were only very weakly related to injury-related variables. The final SEM suggests that pre-injury characteristics impact post-injury outcomes directly, in turn influencing HRQoL after pediatric TBI.

Since our final SEM identifies potential key risk factors that can exacerbate negative outcomes, our findings may serve to better inform patients, caregivers, and clinicians about treatment and care options and can be used in developing preventive and therapeutic programs. Healthcare providers should consider the specific characteristics of children and adolescents (older age, presence of sensory, cognitive, or physical impairments, presence of pre-injury mental health disorders, and neurological and chronic diseases) as well as the characteristics of their parents (lower education) as potential risk factors that can aggravate post-TBI depression, anxiety, PCS, and recovery. Moreover, our results suggest that, if negative post-TBI outcomes arise, targeted and adequate interventions may prevent the deterioration of children’s HRQoL.

## Figures and Tables

**Table 1:** Sociodemographic and injury-related characteristics of children and adolescents after TBI.

Children’s Characteristics	KID (n = 152)	ADO (n = 148)	Total (n = 300)	(Chi-Square, df) *p*-Value
	Pre-Injury Characteristics			
Age (years)				N/A
Mean (SD)	10.6 (1.40)	15.2 (1.47)	12.9 (2.72)	
Median (Range)	10.5 (8.0–12.9)	14.9 (13.0–17.9)	12.9 (8.0–17.9)	
Sex N (%)				(0.44, 1) 0.508
Female	58 (38.2)	62 (41.9)	120 (40.0)	
Male	93 (61.2)	85 (57.4)	178 (59.3)	
Missing	1 (0.7) *	1 (0.7)	2 (0.7)	
Education (of both parents) N (%)				(0.91, 1) 0.340
University	97 (63.8)	92 (62.2)	189 (63.0)	
Other than university	44 (28.9)	53 (35.8)	97 (32.3)	
Missing	11 (7.9)	3 (2.0)	14 (4.7)	
Employment (of both parents) N (%)				(0.86, 1) 0.353
Employed (>35 h/week)	129 (84.9)	133 (89.9)	262 (87.3)	
Other than >35 h/week	18 (11.8)	13 (8.8)	31 (10.3)	
Missing	5 (3.3)	2 (1.4)	7 (2.3)	
Previous TBI N (%)				(0.01, 1) 0.941
No	126 (82.9)	119 (80.4)	245 (81.7)	
One or more TBIs	26 (17.1)	24 (16.2)	50 (16.7)	
Missing	0 (0.0)	5 (3.4)	5 (1.7)	
Prior cognitive, sensory, or physical impairment N (%)				(0.83, 1) 0.362
No	112 (76.7)	102 (68.9)	214 (71.3)	
Yes	40 (26.3)	46 (31.1)	86 (28.7)	
Neurological disease N (%)				(0.39, 1) 0.535
No	143 (94.1)	131 (88.5)	274 (91.3)	
Yes	9 (5.9)	11 (7.4)	20 (6.7)	
Missing	0 (0.0)	6 (4.1)	6 (2.0)	
Chronic disease N (%)				(0.55, 1) 0.457
No	116 (76.3)	103 (69.6)	219 (73.0)	
Yes	36 (23.7)	39 (26.4)	75 (25.0)	
Missing	0 (0.0)	6 (4.1)	6 (2.0)	
Treatment for mental health disorder N (%)				(2.64, 1) 0.104
No	137 (90.1)	130 (87.8)	267 (89.0)	
Yes ***	9 (5.9)	17 (11.5)	26 (8.7)	
Missing	6 (3.9)	1 (0.7)	7 (2.3)	
	Injury-Related Characteristics			
TBI severity N (%)				(0.57, 1) 0.452
Mild	106 (69.7)	109 (73.6)	215 (71.7)	
Moderate or severe	46 (30.3)	39 (26.4)	85 (28.3)	
Imaging finding (lesion) N (%)				(0.05, 1) 0.819
No	105 (69.1)	99 (66.9)	204 (68.0) **	
Yes	43 (28.3)	43 (29.1)	86 (28.7%)	
Missing	4 (2.6)	6 (4.1)	10 (3.3)	
Retrograde amnesia N (%)				(12.64, 1) <0.001
No	124 (81.6)	93 (62.8)	217 (72.3)	
Yes	25 (16.4)	50 (33.8)	75 (25.0)	
Missing	3 (2.0)	5 (3.4)	8 (2.7)	
Loss of consciousness N (%)				(4.09, 1) 0.043
No	111 (73.0)	92 (62.2)	203 (67.7)	
Yes	40 (26.3)	55 (37.2)	95 (31.7)	
Missing	1 (0.7)	1 (0.7)	2 (0.7)	
Neurosurgical intervention following injury N (%)				(0.32, 1) 0.571
No	127 (83.6)	120 (81.1)	247 (82.3)	
Yes	24 (15.8)	27 (18.2)	51 (17.0)	
Missing	1 (0.7)	1 (0.7)	2 (0.7)	
Time since injury (years)				
Mean (SD)	4.2 (2.55)	4.9 (2.95)	4.5 (2.78)	N/A
Median (Range)	3.7 (0.2, 9.4)	4.5 (0.2, 10.2)	4.1 (0.2, 10.2)	
Missing	0	1	1	

Note. Significance tests: Chi-square tests—categorical data, N/A = not available. Missing—reported if any values in the variable are missing. * One child in the KID group was reported as Diverse; they were categorized as missing in the model. Education: the highest achieved of both parents. Employment: the longest of both parents. ** In 162 cases (54.0%) no CT or MRI was undertaken. *** One child in the KID group and seven children in the ADO group had reported mood symptoms. Due to the low proportions of different types of chronic diseases in our sample, we used a dichotomized variable for recording the presence of chronic diseases (Yes/No). Chronic disease types included allergies (n = 14, 4.7%), mental, neurological, and behavioral diseases (n = 11, 3.7%), respiratory tract diseases (n = 10, 3.3%), hematological and immunological diseases (n = 6, 2%), musculoskeletal and connective tissue diseases, skin diseases, endocrine, nutritional and metabolic diseases, visual and hearing impairment, diseases of the heart and circulatory system, diseases of the gastrointestinal or urogenital tract, or other diseases (n = 23, 7.7%). The presence of chronic disease was mentioned by 11 children (3.7%) without specifying the type of disease.

**Table 2:** Summaries of post-injury outcome measures.

Post-Injury Outcome Measures	KID (n = 152)	ADO (n = 148)	Total (n = 300)	(Test Statistic, df) *p*-Value
KOSCHI N (%)				(8.55, 1) 0.004
3a, 3b, 4a, 4b	8 (5.3)	23 (15.5)	31 (10.3)	
5a, 5b	144 (94.7)	125 (84.5)	269 (89.7)	
PCSI ª				
Mean (SD)	5.0 (5.48)	19.8 (18.02)	N/A	N/A
Median (Range)	3.0 (0–27)	13.5 (0–75)		
Missing	19	22		
GAD-7				(0.85, 1) 0.358
Mean (SD)	3.6 (3.07)	3.4 (3.56)	3.5 (3.32)	
Median (Range)	3.0 (0–13)	2.0 (0–17)	2.0 (0–17)	
Missing	5	2	7	
PHQ-9				(1.73, 1) 0.188
Mean (SD)	3.9 (3.36)	4.7 (4.19)	4.3 (3.81)	
Median (Range)	3.0 (0–17)	4.0 (0–21)	3.0 (0–21)	
Missing	5	2	7	
GAD-7 (as categorical) N (%)				N/A
None or minimal anxiety (0–4)	98 (64.5)	110 (74.3)	208 (69.3)	
Mild anxiety (5–9)	43 (28.3)	24 (16.2)	67 (22.3)	
Moderate to severe anxiety (≥10)	6 (3.9)	12 (8.1)	18 (6.0)	
Missing	5 (3.3)	2 (1.4)	7 (2.3)	
PHQ-9 (as categorical) N (%)				N/A
None or minimal depression (0–4)	98 (64.5)	89 (60.1)	187 (62.3)	
Mild depression (5–9)	38 (25.0)	37 (25.0)	75 (25.0)	
Moderate to severe depression (≥10)	11 (7.0)	20 (13.5)	31 (10.3)	
Missing	5 (3.3)	2 (1.4)	7 (2.30)	

Note. ª in the model, a standardized score was used due to different scales for each age group. Tests: Kruskal–Wallis test—continuous data. Chi-square test—categorical data, N/A = not available.

**Table 3:** QOLIBRI-KID/ADO summaries (subscales and total scores).

QOLIBRI-KID/ADO	KID (n = 152)	ADO (n = 148)	Total (n = 300)	(Chi-Square, df) *p*-Value
Total score				(10.39, 1) 0.001
Mean (SD)	76.9 (10.71)	72.6 (11.46)	74.8 (11.27)	
Median (Range)	76.4 (43.6–96.4)	73.6 (27.1–93.6)	75.7 (27.1–96.4)	
Missing	0	3	3	
Cognition				(18.78, 1) <0.001
Mean (SD)	78.6 (11.64)	71.7 (14.29)	75.2 (13.45)	
Median (Range)	78.6 (42.9–100)	75.0 (25–100)	75.0 (25–100)	
Self				(52.54, 1) <0.001
Mean (SD) Missing	86.3 (11.83)	73.6 (16.41)	80.1 (15.61)	
Median (Range)	90.0 (40–100)	75.0 (15–100)	85.0 (15–100)	
Missing	0	1	1	
Daily life and autonomy				(11.37, 1) <0.001
Mean (SD) Missing	88.7 (10.20) 0	84.5 (12.24) 2	86.6 (11.42) 2	
Median (Range)	92.9 (42.9–100)	89.3 (28.6–100)	89.3 (28.6–100)	
Missing	0	2	2	
Social relationships				(13.04, 1) <0.001
Mean (SD)	84.0 (12.24)	79.3 (12.85)	81.7 (12.75)	
Median (Range)	87.5 (37.5–100)	79.2 (29.2–100)	83.3 (29.2–100)	
Emotions				(0, 1) 0.997
Mean (SD)	52.6 (25.02)	53.4 (23.08)	53.0 (24.05)	
Median (Range)	56.3 (0–100)	50.0 (6.3–100)	53.1 (0–100)	
Physical problems				(1.06, 1) 0.303
Mean (SD)	61.9 (23.47)	65.2 (19.58)	63.5 (21.66)	
Median (Range)	62.5 (0–100)	66.7 (8.3–100)	66.7 (0–100)	

Note. Tests: Kruskal–Wallis. Missing—reported if any values in the variable are missing. QOLIBRI-KID/ADO domains: Cognition: 7 items, Self: 5 items, Daily life and autonomy: 7 items, Social relationships: 6 items, Emotions: 4 items, Physical problems: 6 items. Range 0–100, a higher score indicates better HRQoL.

**Table 4:** QOLIBRI-KID/ADO, GAD-7, PHQ-9, PCSI scores, and parental education.

	University (n = 189)	Other than University (n = 97)	Missing (n = 14)	(χ^2^, df) *p*-Value
QOLIBRI-KID/ADO Total				(3.07, 1) 0.080
Mean (SD)	75.6 (10.73)	72.5 (12.36)	78.4 (8.32)	
Median (Range)	75.7 (47.9–96.4)	73.6 (27.1–95)	80.4 (62.1–90)	
Missing	1	2	0	
PHQ-9				(7.95, 1) 0.005
Mean (SD)	3.9 (3.67)	5.2 (4.01)	2.8 (2.64)	
Median (Range)	3.0 (0–21)	4.0 (0–17)	2.0 (0–9)	
Missing	1	1	5	
GAD-7				(5.74, 1) 0.017
Mean (SD)	3.1 (3.03)	4.3 (3.74)	2.9 (3.06)	
Median (Range)	2.0 (0–17)	3.0 (0–16)	2.0 (0–10)	
Missing	1	1	5	
PCSI Total (log z-transformed)				(3.15, 1) 0.076
Mean (SD)	−0.1 (0.95)	0.2 (1.08)	−0.2 (1.07)	
Median (Range)	0.0 (−2.6–2.1)	0.4 (−2.6–2.1)	0.1 (−1.6–1.7)	
Missing	18	21	2	

Note. Kruskal–Wallis test. Due to different scales for PCSI, the PCSI log z-transformed score was used for testing differences between education groups. Missing—reported if any values in the variable are missing.

**Table 5:** Model fitting of measurement and empirical models.

	Measurement and SEMs for QOLIBRI-KID/ADO–FIML Method								
Model Description	Fit Statistics								
	χ^2^	df (Ratio χ^2^/df)	*p*-Value	SRMR	RMSEA	RMSEA 90% CI	GFI	CFI	Difference between Models χ^2^ diff, df, *p*-Value
1. Initial measurement model (all variables in)	571.2	246 (2.3)	<0.0001	0.0849	0.0664	[0.0593, 0.0735]	0.8598	0.8290	
2. Revised measurement model without non-significant paths	452.2	164 (2.8)	<0.0001	0.0887	0.0765	[0.0682, 0.0850]	0.8664	0.8351	
3. First SEM	456.0	165 (2.8)	<0.0001	0.0900	0.0767	[0.0683, 0.0851]	0.8659	0.8334	3.8, 1 df, 0.051 (Model 3–Model 2)
4. Revised final SEM *	456.2	166 (2.7)	<0.0001	0.0901	0.0763	[0.0680, 0.0847]	0.8660	0.8339	0.2, 1 df, 0.655 (Model 4–Model 3) 4.0, 2 df, 0.135 (Model 4–Model 2)

Note. * pathway “Injury-related characteristics” to “TBI-specific HRQoL” removed. Chi-squared difference between the revised SEM and revised measurement model is 4.0 with df = 2, *p* = 0.135. This suggests that the revised SEM accounts for the observed covariances between the factor variables in the structural portion of the model and the revised SEM is not different from the revised measurement model. Χ^2^ = chi-square statistic, df = degrees of freedom, SRMR = standardized root mean square residual, RMSEA = root mean square error of approximation, GFI = goodness-of-fit index, CFI = comparative fit index.

**Table 6:** Properties of the revised model.

Construct and Indicators	Standardized Loading	t ^a^	Reliability	Variance Extracted Estimate
Pre-injury characteristics			0.49 ^b^	0.16
Age	0.24	3.31	0.06	
Education	0.30	4.04	0.09	
Sensory, cognitive or physical impairment	0.36	5.21	0.13	
Neurological disease	0.22	2.87	0.05	
Chronic disease	0.49	6.77	0.24	
Treatment for mental health disorder	0.61	8.87	0.37	
Injury-related characteristics			0.83 ^b^	0.57
Imaging finding (lesion)	0.86	36.97	0.75	
Neurosurgical intervention	0.70	20.65	0.49	
LOC	0.43	8.60	0.19	
Severity of injury	0.92	42.48	0.84	
Post-injury outcome			0.56 ^b^	0.38
PCSI	0.36	5.98	0.13	
KOSCHI	−0.24	−4.00	0.06	
PHQ-9	0.88	26.26	0.78	
GAD-7	0.75	21.39	0.57	
TBI-specific HRQoL (QOLIBRI-KID/ADO)			0.82 ^b^	0.45
Cognition	0.82	30.63	0.67	
Self	0.72	21.53	0.52	
Daily life and autonomy	0.79	27.56	0.62	
Social relationships	0.76	24.64	0.57	
Emotions	0.36	6.46	0.13	
Physical problems	0.41	7.74	0.17	

Note. ^a^ All *t*-tests were significant at *p* < 0.01. ^b^ Denotes composite reliability. The average variance is 0.39. The variance extracted estimate is calculated as the sum of squared factor loadings over the sum of squared factor loadings and the sum of error terms.

## Data Availability

The data presented in this study are available on request from the corresponding authors. Data are not publicly available for reasons of data protection.

## Appendix A. Instruments and Measures

### Appendix A.1. Post-Injury Outcomes

#### Appendix A.1.1. Kings Outcome Scale for Childhood Head Injury (KOSCHI)—Investigator Rated

Recovery as well as the burden of disability caused by a TBI is rated using the KOSCHI [46] with five categories: 1. Death, 2. Vegetative state, 3. Severe disability, 4. Moderate disability, and 5. Good recovery. In this study, only children from recovery categories three to five were included. Each category is split into two subcategories.

#### Appendix A.1.2. Post-Concussion Symptom Inventory (PCSI-SR8/SR13)—Self-Reported by Children and Adolescents

The PCSI [47] is a concussion symptom scale for children and adolescents. It consists of two age-based versions: PCSI-SR8 (17 items answered on a 3-point Guttman scale): 8–12 years, and PCSI-SR13 (21 items using a 7-point Guttman scale): 13–18 years. The tools evaluate the symptoms at baseline as well as PCS retrospectively on four scales (Physical, Emotional, Cognitive, and Sleep/Fatigue). A Retrospective Adjusted Post-Injury Difference (RAPID) score characterizing PCS severity is provided. In this paper, self-reported post-injury versions (PCSI-SR8: 17 items, using a 3-point Guttman scale; PCSI-SR13: 21 items, using a 7-point Guttman scale) were analyzed; a higher score indicates a higher degree of symptoms post-injury. As scores differed for the age groups, standardized continuous scores were used (mean (M) = 0, standard deviation (SD) = 1) to minimize information loss. Scores above or below 1 SD were considered cut-off points for severity of PCS.

#### Appendix A.1.3. Patient Health Questionnaire 9 (PHQ-9)—Parent Rated

The PHQ-9 [48] assesses depression in the previous two weeks according to the Diagnostic and Statistical Manual of Mental Disorders, Fifth Edition (DSM-5) [48]. Total scores range from 0 to 27; a higher score indicates more depressive symptoms. Total scores are calculated by summing the responses on the individual items, coded “0” (not at all) to “3” (nearly every day). Total scores between “1” and “4” are considered minimal depression [48], scores of or above 5, 10, 15, and 20 represented mild, moderate, moderately severe, and severe depression, respectively.

#### Appendix A.1.4. Generalized Anxiety Disorder-7 (GAD-7)—Parent Rated

The GAD-7 [49] assesses symptoms of anxiety according to the DSM-5 [49]. Total scores are calculated by summing the responses on the individual items, coded “0” (not at all) to “3” (nearly every day). The total score ranges from 0 to 21 with higher values indicating greater disturbance. Total scores of or above 5, 10, and 15 represent mild, moderate, and severe impairment, respectively [49].

### Appendix A.2. TBI-Specific HRQoL Measure

#### Quality of Life after Traumatic Brain Injury in Kids/Adolescents (QOLIBRI-KID/ADO)—Self-Reported by Children and Adolescents

The QOLIBRI-KID/ADO [50] is a novel TBI-specific age-adapted HRQoL questionnaire for children (KID) aged 8–12 and adolescents (ADO) aged 13–17 years allowing self- and/or proxy-assessment. The items refer to now including the last week and are self-rated on a 5-point Likert scale (“not at all”, “slightly”, “moderately”, “quite”, “very”), with higher scores indicating higher HRQoL. Items are rated in terms of being either satisfied with or bothered by the consequences of a TBI. It contains 35 items distributed between six subscales: Cognition, Self, Daily life and autonomy, Social relationships, Emotions, and Physical problems. As in the adult version [71], scores are calculated for each of the QOLIBRI-KID/ADO subscales, as well as a total score. Scores are expressed on a scale from 0 to 100, prorated if up to one third of scale values are missing, and a higher score indicates better HRQoL.
